# Expression profiles of metallothionein-I/II and megalin/LRP-2 in uterine cervical squamous lesions

**DOI:** 10.1007/s00428-020-02947-w

**Published:** 2020-10-21

**Authors:** Hrvoje Jakovac, Nikola Stašić, Maja Krašević, Nives Jonjić, Biserka Radošević-Stašić

**Affiliations:** 1 Department of Physiology and Immunology, Medical Faculty, University of Rijeka, B. Branchetta 20, 51 000 Rijeka, Croatia; 2 Teaching Institute of Public Health, Primorsko-goranska County, Medical Faculty, University of Rijeka, Rijeka, Croatia; 3 Department of Pathology, Medical Faculty, University of Rijeka, Rijeka, Croatia

**Keywords:** Akt1/protein kinase B phosphorylation, Biomarkers, CIN lesions, low-density lipoprotein receptor–related protein-2, Metallothionein-I/II, Tumour microenvironment

## Abstract

Metallothioneins (MTs) are phylogenetically old cysteine-rich proteins, which are implicated in a variety of physiological and pathological processes. Their growth-regulating, anti-apoptotic and anti-inflammatory functions have been attributed not only to intracellular free radical scavenging and to zinc and copper regulation but also to the ability of secreted MT to bind on surface lipoprotein receptor-megalin/LRP2, which enables the endocytosis of MT-I/II and a wide range of other functionally distinct ligands. In the present study, we analysed the expression pattern of both proteins in 55 cases of premalignant transformation of cervical squamous cells, i.e. in low- and high-grade squamous intraepithelial lesion (LSIL and HSIL). The data showed that in LSIL (cervical intraepithelial neoplasia CIN1; *N* = 25) MTs were present only in basal and parabasal cells and that megalin was only weakly expressed. In HSIL (CIN2; *N* = 15 and CIN 3/carcinoma in situ; *N* = 15), however, overexpression and co-localization of MT with megalin were found in the entire hyperplastic epithelium. Moreover, megalin immunoreactivity appeared on the glandular epithelium and vascular endothelium, as well as on lymphatic cells in stroma. Besides, multiple megalin-positive cells expressed phosphorylated Akt1, implying that MT- and/or megalin-dependent prosurvival signal transduction pathways might contribute to the development of severe cervical dysplasia. The data emphasize the diagnostic power of combined MT/megalin analysis in pre-cancer screening.

**Electronic supplementary material:**

The online version of this article (10.1007/s00428-020-02947-w) contains supplementary material, which is available to authorized users.

## Introduction

Metallothioneins (MTs) are a family of phylogenetically highly conserved, cysteine-rich, small (< 10 kDa) proteins, which are characterized by high affinity for d10 electron configuration metals, including essential (Zn and Cu) and non-essential (Cd and Hg) trace elements [1, 2]. They were initially considered the mediators of cellular detoxification of heavy metals such as Cd, Hg, Cu and Ag [3], but due to the high kinetic mobility of essential metals and regulation of zinc availability to numerous cellular enzymes, signalling proteins and transcription factors, they are also involved in the regulation of basic cellular processes, such as gene expression, differentiation, proliferation and apoptosis [1, 4]. In addition, due to their high cysteine content, MTs protect cellular macromolecules from highly reactive compounds during oxidative stress and other types of tissue injuries, acting as potent scavengers of reactive oxygen species (ROS), such as hydrogen peroxide (H_2_O_2_), superoxide (O_2_–), nitric oxide (NO) and hydroxyl (OH) radicals [5].

Importantly, exogenous MTs may also interact with multiligand, scavenger receptors belonging to the family of low-density lipoprotein receptor (LDL-R)–related proteins (LRPs), such as LRP-2/megalin and LRP-1/CD91. These endocytic receptors consequently mediate the cellular uptake, proteolysis and gene expression of MTs, or induce the activation of new, LRP-related molecular signalling pathways [6, 7]. In this context, it has been shown that the MT/LRP2 interactions mediate numerous neuroprotective and neuroregenerative effects particularly in the central and peripheral nervous system [8–14].

Owing to their features, both MTs and LRPs have been extensively investigated as diagnostic and prognostic markers of various types of cancer and factors implicated in processes of oncogenesis and tumour progression [16–21]. The data were related with the intercourse of MTs with zinc-dependent enzymes and transcription factors important for cell cycle regulation and proliferation control, such as p53, nuclear factor κB (NFκB) and PKC μ [20] and with the ability of MTs to protect cells against DNA damage, oxidative stress and apoptosis [2, 17, 18, 21], as well as with hypermethylation of the MT-promoter or mutation of other genes [22]. It was emphasized that in cancer cells, metallothionein’s protective functions might stimulate tumour progression and malignancy, but the results varied and depended on the type, differentiation status and proliferative index of tumours, as well as on the type of analysed MT isoforms [15, 18, 21]. Besides, only in few studies, the possible contribution of MT/megalin interactions to the process of carcinogenesis has been investigated [23].

In an attempt to enlarge still limited data available on the role of MTs in uterine cervical squamous lesions [24–26], in the present study, we made immunohistochemical profiling of MT-I/II and megalin expression in different types of pre-invasive intraepithelial neoplasia-LSIL(CIN1) and HSIL (formerly subdivided into CIN2 and CIN3/carcinoma in situ) that in some cases progress to invasive cervical cancer, which represents the fourth leading cause of cancer-related deaths in women worldwide [27].

## Patients and methods

### Clinical material

Cervical tissue samples were obtained from 55 patients by cervical biopsy, taken by gynaecologists at an area of colposcopic abnormality and from 5 patients undergoing total abdominal hysterectomy for benign disease of the corpus uteri with no evidence of prior cervical cytological abnormality. Formalin-fixed and paraffin wax–embedded tissue was stained by haematoxylin and eosin and examined by two pathologists. After independent reviews, the specimens were routinely classified as normal (*N* = 5), LSIL/CIN1 (*N* = 25) and HSIL (*N* = 30), subdivided into CIN2 (*N* = 15) and CIN3/squamous cell carcinoma in situ (CIS) (*N* = 15). All subjects gave their informed consent for inclusion before they participated in the study. The study was conducted in accordance with the Declaration of Helsinki, and the protocol was approved by the Ethics Committee of Clinical Hospital Centre and Medical Faculty in Rijeka.

### Immunohistochemistry

Immunohistochemical labelling of MT I + II and megalin proteins was performed on paraffin-embedded tissues using DAKO EnVision+System, Peroxidase (DAB) kit according to the manufacturer’s instructions (DAKO Cytomation, USA) as previously described [14].

Briefly, slides were incubated with peroxidase block to eliminate endogenous peroxidase activity. After washing, mouse monoclonal anti-MT I + II IgG1 (clone E9; Dako Cytomation, USA; diluted 1:50 with 1% BSA in PBS) that reacts against a conserved epitope of murine and human MT-1 and MT-2 isoforms [24, 26] or rabbit polyclonal anti-megalin IgG (H-245, Santa Cruz Biotechnology, USA; diluted 1:200 with 1% BSA in PBS) were added to the tissue samples and incubated overnight at 4 °C in a humid environment, followed by 45-min incubation with peroxidase-labelled polymer conjugated to goat anti-mouse or anti-rabbit immunoglobulins containing carrier protein linked to Fc fragments to prevent nonspecific binding. The immunoreaction product was visualized by adding substrate chromogen (DAB) solution. Tissues were counterstained with haematoxylin, dehydrated through graded ethanol, and mounted using Entellan (Sigma-Aldrich, Germany). The specificity of the reaction was confirmed by substitution of antigen-specific antibody with mouse irrelevant IgG1 kappa immunoglobulin (clone DAK-G01; Dako, USA), used in the same conditions and dilutions as a primary antibody. As positive controls for the sensitivity of procedure, we used regenerating liver tissue obtained from partially hepatectomized mice for MT I/II [28] and choroid plexus tissue obtained from cuprizone-treated mice for megalin [14]. The microphotographs were taken and examined under an Olympus BX51 light microscope (Olympus, Japan).

### Immunofluorescence

Single and double labelling of MT-I/II and megalin were performed on paraffin-embedded cervical tissue slides by the use of mouse anti-MT I + II IgG1 (clone E9; Dako Cytomation, USA; diluted 1:50), rabbit anti-megalin IgG (H-245, Santa Cruz Biotechnology, USA; diluted 1:50), rabbit anti-CD3 IgG (Abcam, UK, 1:100) and mouse anti-AKT1 (phospho-T308) IgG1 (Abcam, UK, 1:50).

Tissue sections were submitted to heat-induced antigen retrieval and nonspecific binding was blocked by one-hour incubation with 1% BSA in PBS containing 0.001% NaN3 at room temperature. Immunocomplexes were visualized by secondary antibodies Alexa Fluor goat anti-mouse IgG1 555 nm (Molecular probes, USA, 1:500) and Alexa Fluor donkey anti-rabbit IgG 488 nm (Molecular Probes, USA, 1:300). Secondary antibodies were diluted in blocking solution and incubated with tissue sections in dark for 1 h at room temperature in a humid environment. Nuclei were visualized with 4′, 6-diamidino-2-phenylindole, dihydrochloride (DAPI, Molecular Probes, USA,). Images were captured on Olympus imaging system BX51 equipped with a DP71CCD camera (Olympus, Tokyo, Japan) and the CellF imaging software was used. Antibodies used in the study are listed in the supplementary material (Table 1 supplement).

**Fig. 1:**
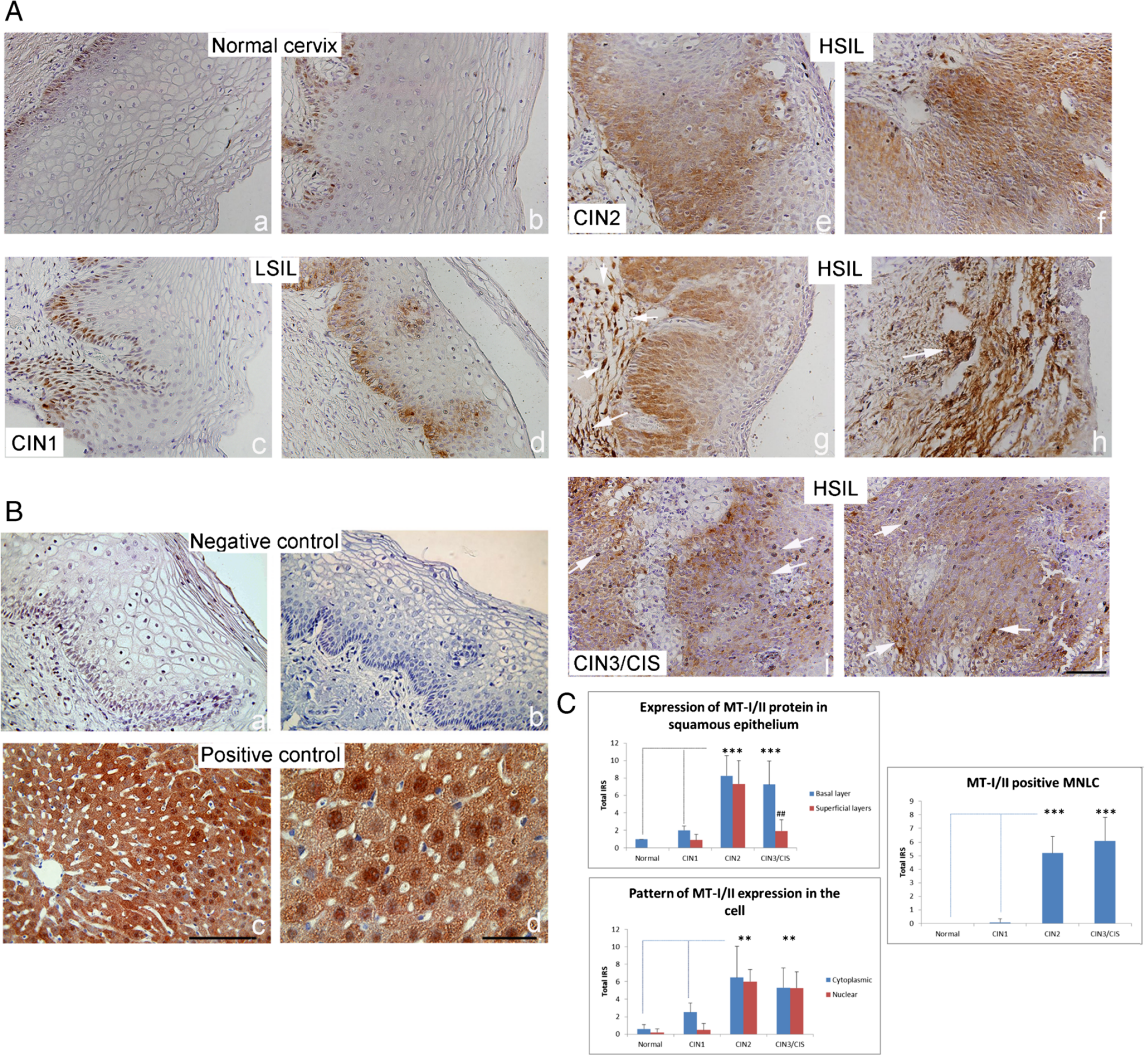
MT-I/II immunoreactivity in low- and high-grade squamous intraepithelial lesions. (A) Representative immunohistochemical pictures show staining with anti-MT-I/II antibody in paraffin-embedded sections of the cervical tissue samples, classified as normal cervix (a, b), LSIL (CIN1) (c, d) and HSIL, subdivided as CIN2 (e–h) and CIN3/CIS (i, j). (B) Negative (isotype-matched) control—staining of cervical tissue by mouse irrelevant IgG1 kappa immunoglobulin (a, b); Positive control—staining of murine hepatocytes after 1/3 partial hepatectomy by anti-MT-I/II antibodies (c, d) [28]. Scale bars 50 μm (A a–j; B a–c) and 20 μm (B d). (C) MT-I/II immunoreactivity in different SIL/CIN categories (expressed as total immunoreactive score (IRS) in squamous epithelium, in specific cell compartment and in mononuclear lymphatic cells (MNLC). IRS was calculated by taking the product of staining intensity (ranged from 0 to 3) and percentage positivity (ranged from 1 to 4). Values are expressed as mean ± SE. *p < 0.05, **p < 0.01 and ***p < 0.001 in comparison with basal layer in the intact cervix; #*p* < 0.05, ##*p* < 0.01 comparison of the basal and superficial layer in each group

### Semi-quantitative analysis and immunohistochemical staining quantification

The intensity and distribution of MT and megalin immunostaining were evaluated in sections of the cervix stained by anti-MT I + II or by anti-megalin antibodies after photographing under a light microscope (magnification × 400, Olympus BX51 Microscope). The analysis was independently made by 2 evaluators in specific tissue areas (basal and parabasal layers of normal/dysplastic epithelium, glandular epithelium, mononuclear lymphatic cells) and in specific cellular compartment (cytoplasm or nuclei).

Immunohistochemistry results were evaluated on the basis of staining intensity and percentage positivity as described by Jawanjal et al. [29]. Briefly, the intensity of protein expression was scored on the scale of 0–3 positivity (‘0’, negative staining; ‘1’, weak; ‘2’, moderate and ‘3’, intense positive staining), while percentage positivity of stained cells was ranged from 1 to 4+ (‘0’, complete absence; ‘1’, < 5%; ‘2’, 5–20%; ‘3’, 21–50%; ‘4’, > 50% positive stained cells). The total immunoreactive score (IRS) was calculated by taking the product of both intensity and percentage positivity value. The data are presented as average IRS ± standard error of the mean (SEM) on scale, ranging from 0 to 12. For analytical purposes, the IRS scores were further clustered into three grades: 0–1 = absent, 2–5 = low expression and 6–12 = high expression.

In addition, in 3 cervical biopsy samples per grade of CIN group the immunohistochemical staining quantification of MT-I/II and megalin expression was performed using Image J software, as we previously described [14]. For this purpose captured images were converted to 16-bit images based on grey-scale with different grey intensity range, depending on the strength of immunohistochemical signals. Digital background subtraction was done and intensities were inverted in order to achieve positive correlation of staining intensity and brightness as a grey intensity. The threshold was then manually set so that any background brightness was considered as the value 0, and background signals were completely excluded from the calculation. Regions of interest (ROIs) were arranged to cover the area being analysed and ROI surface size was always equal for each analysed area. Three regions of interest were analysed per field (× 400) in the ten fields per microscopic slide of tissue samples, derived from the intact cervix, LSIL and HSIL. The data were expressed as average grey intensity ± SEM.

### Statistical analysis

The statistical analyses were performed using Statistics software version 12 (StatSoft Inc., Tulsa, OK, USA). The distribution of data was tested for normality using the Kolmogorov-Smirnov test. Differences between groups were assessed with *χ*2 test or with one-way analysis of variance (ANOVA) followed by the post hoc Scheffé test. The level of significance was set at *p* < 0.05.

## Results

### Expression of metallothionein-I/II in LSIL and HSIL

Immunohistochemical analysis of MT-I/II expression was made in cervical tissue samples classified as normal, LSIL (CIN1) and HSIL (subdivided in CIN2 and CIN3/CIS). The data are presented in Fig. 1 and on Table 2 (in supplement), which shows the total IRS of MT-I/II expression obtained by multiplying the staining intensity of MTs by the percentage of MT-positive cells. They show that in intact cervix the immunoreactivity of MTs was almost negligible (Fig. 1A a, b) and that in LSIL (CIN1), it was limited to basal and parabasal cells of the squamous epithelium at the squamocolumnar junction (Fig. 1A c, d). In HSIL, however, in the majority of CIN2 cases, the intense cytoplasmic and nuclear expressions of MT were found throughout the full thickness of the dysplastic squamous epithelium, both in basal and in superficial layers (Fig. 1A e–g), as well as on several subepithelial stromal cells (arrows on Fig. 1A g, h). In the cases of CIN3/CIS, a prominent MT-I/II staining was observed on the dysplastic epithelium (Fig. 1A i, j), as well as on several infiltrating mononuclear lymphatic cells (MNLC) in stroma (arrows on Fig. 1A i, j). The semi-quantitative analysis also showed that in higher grades of CIN lesions, the total MT-I/II IRS in basal epithelium increased from 2.0 ± 0.5 (CIN1) to 8.3 ± 2.3 (CIN2) and 7.3 ± 2.7 (CIN3/CIS), and that in CIN2, its IRS in the superficial layer (7.3 ± 2.7) was significantly greater than in other types of dysplasia (Fig. 1C; *p* < 0.001).

Moreover, in HSILs increased the nuclear expression of MTs (high IRS was found in 80% of CIN2 and in 53.3% of CIN3/CIS cases) and the infiltration with MT-I/II positive MNLC (high IRS were found in 66.7% and 73.3% of cases, respectively) (Fig. 1C; Table 2 supplement). 

### Megalin immunoreactivity

**Fig. 2:**
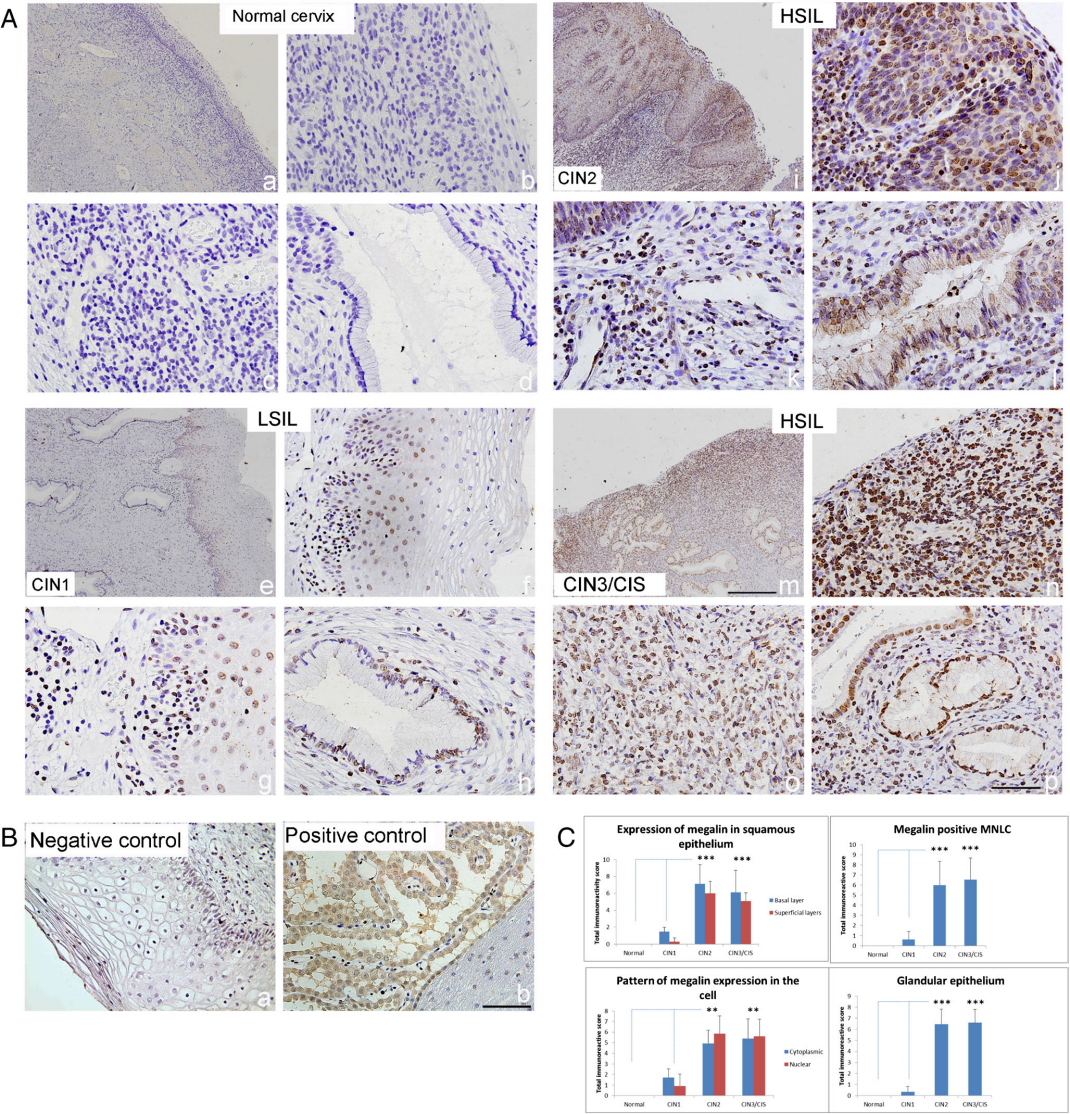
Megalin immunoreactivity in low- and high-grade squamous intraepithelial lesions. (A) Representative immunohistochemical pictures show staining with anti-megalin antibody in paraffin-embedded sections of the cervical tissue samples, classified as normal cervix (a–d), LSIL (CIN1) (c–h) and HSIL, subdivided as CIN2 (i–l) and CIN3/CIS (m–p). (B) Negative (isotype-matched) control—staining of cervical tissue by mouse irrelevant IgG1 kappa immunoglobulin (a, b); Positive control—staining of choroid plexus in cuprizone-treated mice by anti-megalin antibody [14]. Scale bars 100 μm (A a, e, i, m); 50 μm (all other pictures). (C) Megalin immunoreactivity in different SIL/CIN categories (expressed as total immunoreactive score (IRS) in squamous epithelium, in specific cell compartment, in mononuclear lymphatic cells (MNLC) and in glandular epithelium IRS was calculated by taking the product of staining intensity (ranged from 0 to 3) and percentage positivity (ranged from 1 to 4). Values are expressed as mean ± SE. ***p < 0.001 in comparison with findings in intact cervix

Staining with anti-megalin antibody did not result in any immunopositivity in normal cervical tissue samples (Fig. 2A a–d). In LSIL (CIN1), the expression of megalin appeared on some cells in squamous and glandular epithelium (Fig. 2A e–h), but in HSIL (CIN2), it was visible on numerous epithelial cells in dysplastic zone (Fig. 2A i, j) as well as in numerous MNLC in stroma and in glandular epithelium (Fig. 2A k, l). Megalin overexpression became even more visible in the cases of CIN3/CIS on MNLC that infiltrated the stroma (Fig. 2A m, n) and on epithelial cells in several glands (Fig. 2A o, p). In these locations, high scores of cytoplasmic and nuclear megalin expression were found in great number of examined cases of CINs (53.3% and 86.7% in CIN2 and 66.7% and 73.3% in CIN3/CIS) (Table 3; supplement). Besides, the data obtained by semi-quantitative analysis showed that in the cases of CIN2 and CIN3/CIS, the megalin overexpression in basal and superficial layers of squamous and glandular epithelium was significantly greater than that in other types of cervical lesions (Fig. 2C; *p* < 0.001).

Notably, the additional analysis performed by the Image J software confirmed that MT-I/II and megalin immunoreactivities in squamous epithelium were greater in HSILs than in LSILs (Fig. 1 in supplement).

### MT-I/II and megalin expression in HSIL

Since previous, immunohistochemical findings clearly showed that MT-I/II and megalin were overexpressed particularly in HSIL (CIN2 and CIN3/CIS), by double immunofluorescence, we further attempted to visualize the possible co-localization of MT-I/II and megalin in affected cervical regions.

#### CIN2

**Fig. 3:**
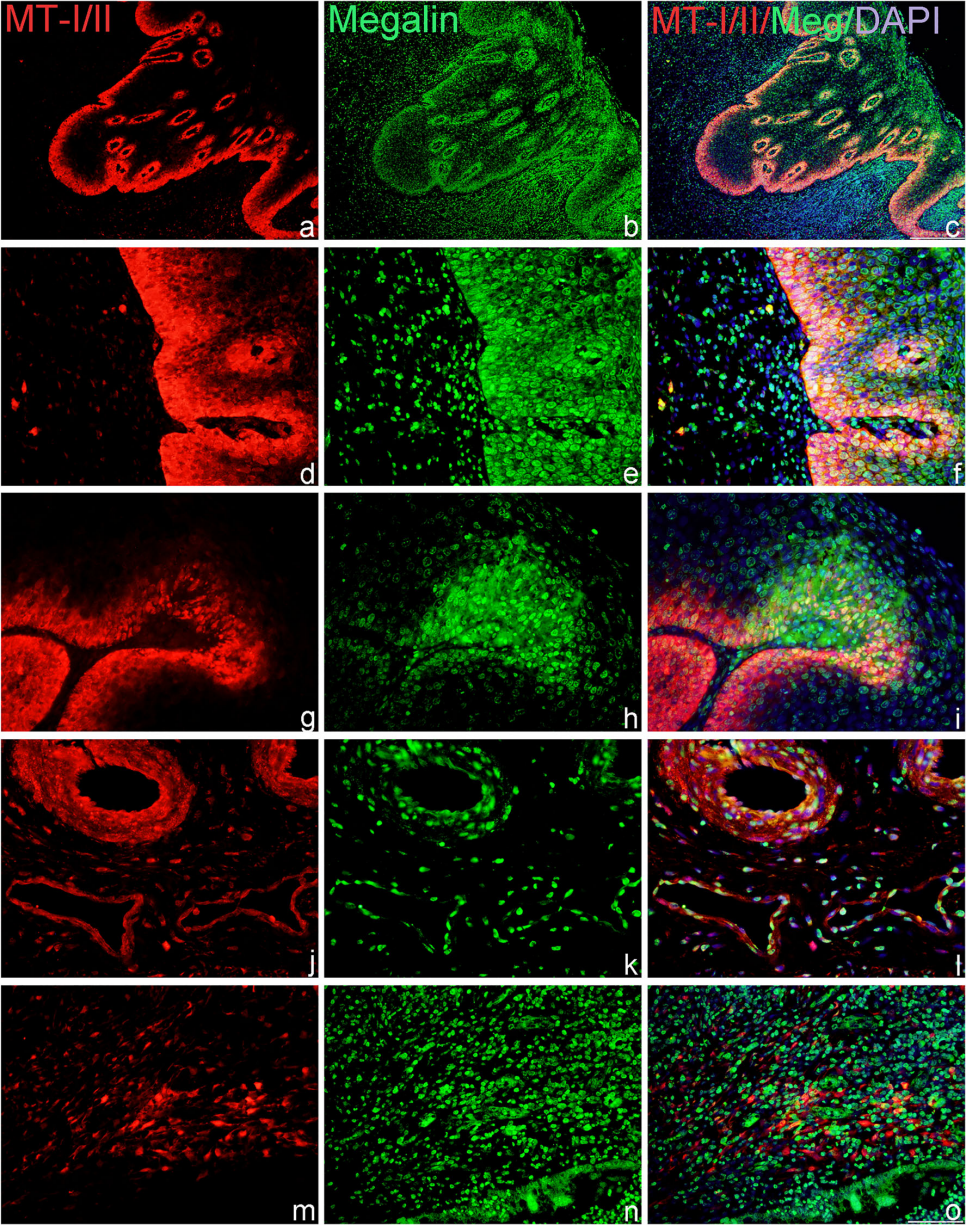
MT-I/II and megalin expression and co-expression in HSIL (CIN2). Cells expressing MTs and megalin were detected by the use of anti-MT-I + II (red staining) and anti-megalin antibodies (green staining) in paraffin-embedded sections of the cervical tissue samples, classified as HSIL/CIN2 lesions. Blue marks DAPI staining of nuclei and yellow marks the overlapping of MT-I + II with megalin. Representative images show findings in squamous epithelium (a–i); in glandular epithelium and vascular endothelium (j–l) and in lymphatic infiltrates in stroma (m–o). Scale bars: 100 μm (a–c) and 50 μm (d–o)

As shown on Fig. 3, immunofluorescent staining confirmed that in HSIL (CIN2), MT-I/II and megalin were expressed in the same areas of dysplastic squamous epithelium (a–f). In addition, in these places (g–i) and around some MT-positive mononuclear cells in stroma, multiple megalin-positive lymphoid cells were found (m–o). The data also showed that in HSIL, the MT-I/II and megalin immunoreactivity were expressed in glandular epithelium and in vascular endothelium and that some of these cells co-expressed both proteins (Fig. 3 j–l).

#### CIN3/CIS

**Fig. 4:**
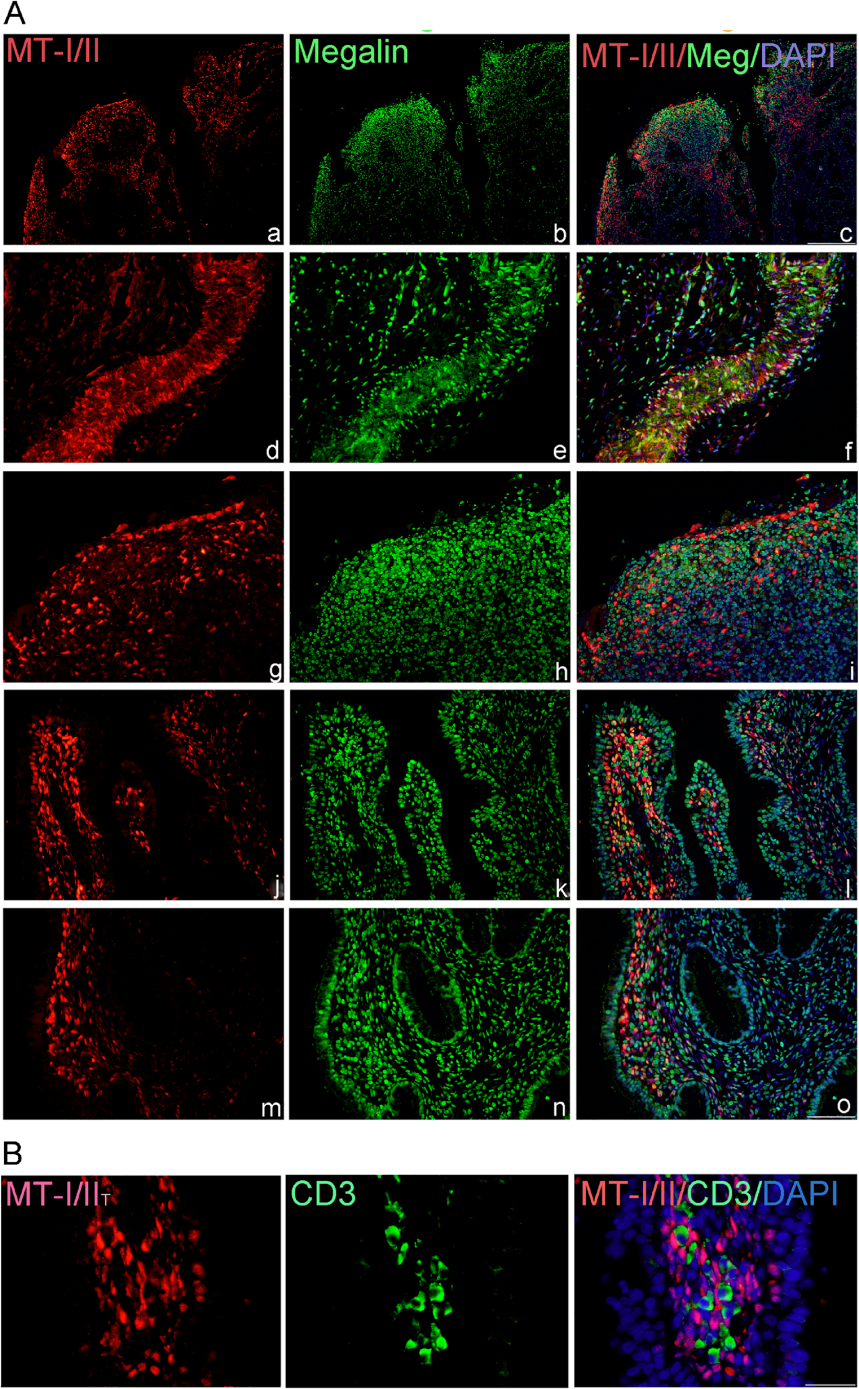
(A) MT-I/II and megalin expression and co-expression in HSIL (CIN3/CIS). Cells expressing MTs and megalin were detected by the use of anti-MT-I + II (red staining) and anti-megalin antibodies (green staining) in paraffin-embedded sections of the cervical tissue samples, classified as CIN3/CIS. Blue marks DAPI staining of nuclei and yellow marks the overlapping of MT-I + II with megalin. Representative images show findings in squamous epithelium (a–i); in glandular epithelium and lymphatic infiltrates in stroma (j–o). Scale bars: 100 μm (a–c) and 50 μm (d–o). B) MT-I/II and CD3 expression in stroma of CIN3/CIS. Representative images show the presence of T lymphocytes (green staining) in the vicinity of MT-I/II-positive cells (red staining). Scale bars 50 μm

Single immunofluorescence for MTs confirmed that in a more aggressive form of cervical lesions (CIN3/carcinoma in situ) MT-I/II was expressed on dysplastic squamous epithelial cells (Fig. 4A a, d, g), as well as on numerous MNLC in stroma (Fig. 4A j, m). Some of MT-positive epithelial cells co-expressed also megalin (Fig. 4A d–f), but on numerous mononuclear cells in stroma (Fig. 4A b, h) and on the glandular epithelium of the endocervix (Fig. 4A k, n) the sole megalin immunoreactivity was found. Immunostaining with anti-CD3 antibodies showed that in the vicinity of MT-I/II, positive cells were present in several T lymphocytes (Fig. 4B).

### Megalin-positive cells express phosphorylated Akt-1/protein kinase B

**Fig. 5:**
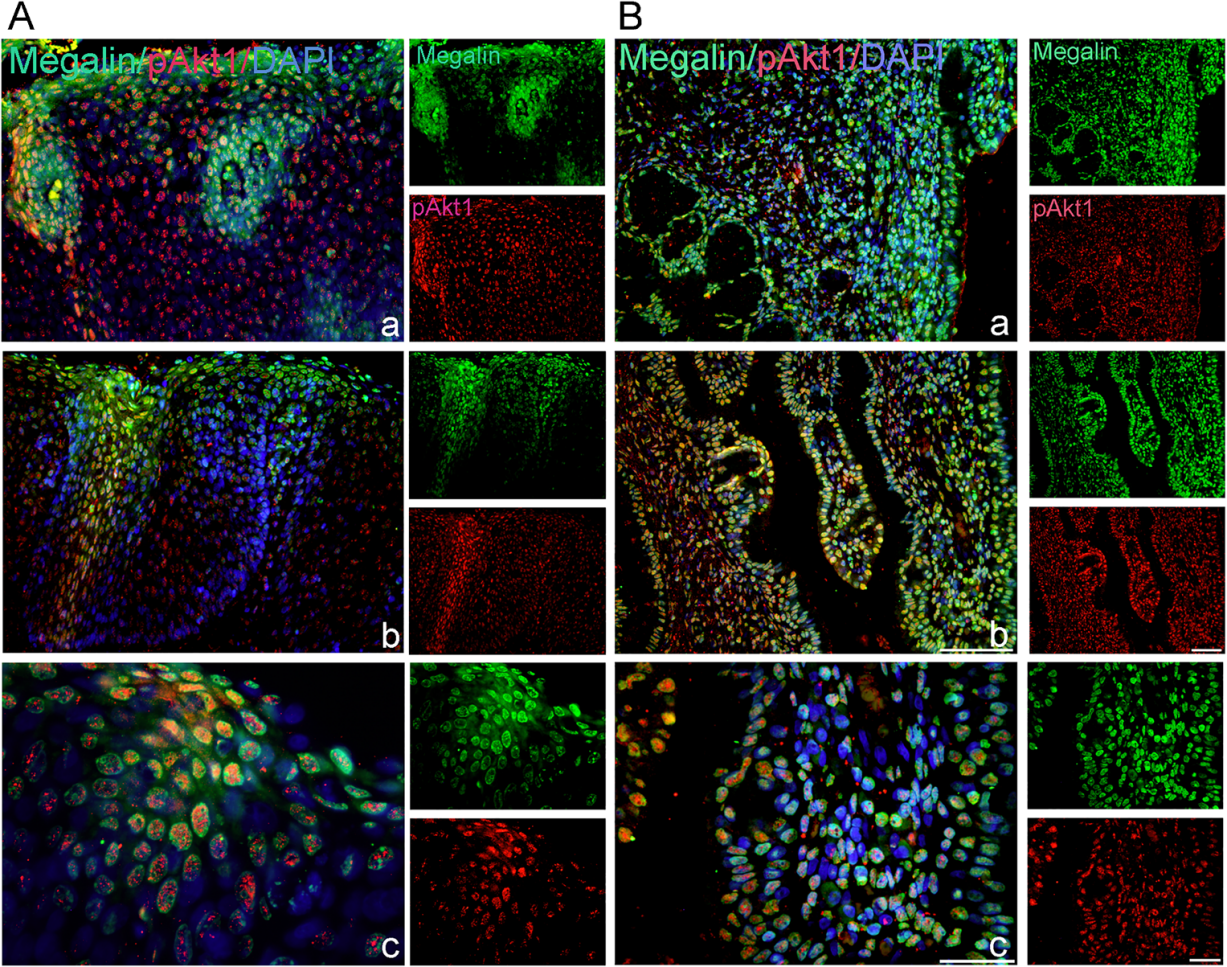
Megalin-positive cells express phosphorylated Akt-1/protein kinase B. Cells expressing megalin (green staining) and phospho-AKT1/protein kinase B (pAkt1) (red staining) were detected by the use of anti-megalin and anti-pAkt1 (phospho-Thr308) antibodies in paraffin-embedded sections of the cervical tissue samples, classified as HSIL (CIN2) (A) or CIN3/CIS (B). Blue marks DAPI staining of nuclei and yellow marks the overlapping of megalin with pAkt1. Scale bars: 50 μm (A a, b; B a–c) and 20 μm (A c)

Since binding of MT-I/II or other ligands to megalin/LRP2 receptor may in target cells induce the activation of serine/threonine-protein kinase-Akt-1**/**protein kinase B signalling cascade [30], in megalin-positive cells, we evaluated also the presence of phosphorylated Akt-1 (pAkt-1; phospho-threonine 308). The data obtained by double immunofluorescence showed that in HSILs several megalin-positive squamous cells (Fig. 5A a–c; B a), lymphocyte-like cells (Fig. 5 B c) and glandular epithelial cells (Fig. 5B a–c) co-express pAkt-1.

## Discussion

In agreement with a number of previous reports, pointing to high MT expression in uterine cervical squamous lesions and in aggressive endometrial adenocarcinomas [24–26, 31], in the current study, we have confirmed that MT-I/II overexpression may be found within the transformation zone of the cervix particularly in the cases of HSILs (CIN2 and CIN3/CIS). These well-known findings are generally related to the pro-proliferative and anti-apoptotic activities of MTs and their intracellular ability to act as a reservoir of metals for numerous cellular proteins, enzymes, DNA and RNA polymerases and transcription factors that require Zn for their biological activity, and as potent scavengers of ROS in conditions of stress, hypoxia and inflammation [2, 15, 16, 18]. It is, however, also emphasized that overexpression of MT-I/II might have both anti-oncogenic and oncogenic potential, since, in initial stages of tumour development, they may suppress mutation, while in tumour cells, they may promote accelerated growth and survival of neoplastic cells preventing the binding of tumour suppressor gene p53 to DNA or inducing a reversible conformation of wild-type p53 to the mutant form by binding zinc ions to cysteinyl residues [16, 18, 31, 32]. Moreover, it was reported that p53 might be directly involved in transcription of MT-1A and MT-2A genes in epithelial breast cancer cells [22].

Our data showing that in cervical microenvironment of severe forms of CINs concomitantly increases the megalin/LRP2 and pAKT1 immunoreactivity suggest that some differentiation and survival-promoting effects of MT-I/II might be obtained through receptor-activated endocytosis of extracellular MTs or through the activation of other megalin-dependent signal transduction pathways that lead to the activation of Src/ErK/Akt/CREB. Since these changes were not observed in normal cervical tissue, nor in LSILs (CIN1), we may speculate that they probably contributed to the better survival of aberrant cells in squamous epithelium and process of oncogenesis, but although similar MT/megalin interactions have been described as important cell signalling pathways mediating neuroprotective and neuroregenerative effects of MTs in the central nervous system (CNS) [7–9, 13, 14, 33–35], so far, only a few reports showed their involvement in carcinogenic processes. Thus, consistent with our data, it has been reported that MT-I/II and megalin are significantly altered in primary CNS lymphoma [23], as well as that melanoma tumours, which express high MT immunoreactivity [36, 37] frequently acquires LRP2/megalin expression [19]. Moreover, the latter study showed that megalin expression in melanoma cells was crucial for cell maintenance, since siRNA-mediated reduction of LRP2/megalin expression in melanoma cells significantly decreased their proliferation and survival rates [19].

Based on these findings, we may speculate that MT-I/II/megalin interactions contributed to the development of severe dysplasia and better survival of malignantly transformed cells in cervical squamous epithelium, but mechanisms need to be elucidated. The hypothesis is, however, supported by the evidence showing that increased levels of extracellular MT in combination with other factors trigger the upregulation of megalin in absorptive epithelia in the kidney [38], as well as in ependymal cell lining the ventricular wall, capillaries and choroid plexus cells in the brain [14, 33, 39]. As extensively reviewed by Atrian and Capdevila, the mechanisms might include both physical interactions of MTs with their receptors and other proteins, as well as metal swap events, related to Zn/Cu exchange reactions and function of MT3 isoform [20]. In accordance with the first proposal, in different neuronal models, it was shown that MT-I/II, internalized by megalin, might itself activate molecular signalling pathways by the release of zinc into the cytoplasm, as well as that binding of MT to megalin might result in the cleavage of a C-terminal intracellular component of megalin and in induction of classical receptor-like signal transduction properties, such as activation of the MAPK-dependent signalling pathways [9, 11, 12] or AKT/Protein kinase B [14]. In this regard, our data need further investigation, but findings of nuclear expression of megalin and pAkt1 (Thr308) in affected cells (Fig. 5) point to potential intramembranous proteolysis of megalin, since these cytosolic fragments contain critical signalling motifs for interaction with a set of cytoplasmic adaptor and scaffold proteins and in some instances enter the nucleus to regulate transcription of target genes [40–43].

In line with current evidence, we, thus, can speculate that megalin-dependent signals induced by binding of MT-I/II or other signals, such as extracellular matrix (ECM) proteins, cell surface components, growth factors, morphogens, and cytokines, contribute to the epithelial–mesenchymal transition (EMT) program and malignant transformation of cervical epithelial cells [44, 45]. Besides, since in HSILs we found that megalin immunoreactivity appeared also on numerous mononuclear lymphoid cells, as well as on vascular endothelium and glandular epithelium (Figs. 2, 3 and 4), i.e. on structures which, in intact cervical tissue and in CIN1, did not express megalin (Fig. 2A a–f), we also underline that megalin/LRP2, as multiligand endocytic receptor, might be involved in uptake and transport of vital nutrients and growth factors required for the survival of activated cells (lipoproteins, albumins, vitamin B_12_ and D binding proteins, ferritin, epidermal growth factor, insulin-like growth factor 1, etc.) [46, 47], as well as in endocytosis and trafficking of ligands that in tumour microenvironment (TME) regulate the processes of innate and adaptive immunity. To our knowledge, the potential effects of megalin-related pathways on these functions have not been emphasized, but it is well known that TME employs multiple mechanisms to switch off the anti-tumour functions of immune cells [48, 49]. MT-I/II-positive macrophage-like cells in stroma (Figs. 3 and 4 A, B) probably contributed to these events since in different types of squamous cell carcinoma and in breast adenocarcinoma, it has been shown that the expression of MT-I/II in cancer-adjacent stroma might reduce the number of CD56- and CD57-positive lymphocytes and contribute to immune system inhibition and tumour progression [32], as well as that zinc and MT might play crucial roles in NK and NKT cell development and functions [50]. Noteworthily, the data also showed that the intensity of the MT immunoreactivity in uterine cervical cancer might be linked to both the depth of the local invasion and the extent of the distant advancement of the disease [25] and that cross-talk between myeloid-derived suppressor cells, macrophages, and dendritic cells may enhance tumour-induced immune suppression and stimulate tumour neovasculature [49]. In this context, our data need further investigations, but to our knowledge, this is the first investigation pointing to the MT/megalin interaction and significance of megalin expression in TME for the development of severe CIN lesions. In conclusion, in this report, we demonstrate that HSIL in dysplastic squamous epithelium raises not only MT-I/II expression but also the expressions of multiligand endocytic receptor-megalin/LRP2, which may through internalization of exogenous MTs or through receptor-accelerated phosphorylation of Akt1/protein kinase B promote the survival of activated cells. In addition, we show that in advanced squamous cervical lesions (CIN2I and CIN3/CIS), megalin expression rises also on several lymphoid, epithelial and endothelial cells, as well as that numerous stromal, macrophage-like cells, expresses MT-I/II immunoreactivity, indicating that these changes may participate in a reprogramming and polarization of the innate cell compartment and contribute to immune system inhibition and tumour progression and dissemination.

Taking together, the data indicate that the combined analysis of MT and megalin may enhance the diagnostic power of each individual marker and improve the preventive and therapeutic strategies for cervical cancer.

## Electronic supplementary material

ESM 1 (DOCX 14 kb)

ESM 2 (DOCX 15 kb)

ESM 3 (DOCX 15 kb)

Fig. 1 Supplement. MT-I/II and megalin expression in cervical squamous epithelium in different SIL/CIN categories. The intensity and distribution of MT and megalin staining were estimated by ImageJ software analysis. The data are expressed as mean grey value ± SE. *p < 0.05, **p < 0.01 and ***p < 0.001 in comparison with basal layer in intact cervix; #p < 0.05, ##p < 0.01 comparison of basal and superficial layer in each group (PNG 158 kb)

High resolution image (TIF 183 kb)

## References

[1] Isani G, Carpene E (2014). Metallothioneins, unconventional proteins from unconventional animals: a long journey from nematodes to mammals. Biomolecules.

[2] Coyle P, Philcox JC, Carey LC, Rofe AM (2002). Metallothionein: the multipurpose protein. Cell Mol Life Sci.

[3] Margoshes M, Vallee B (1957). A cadmium protein from equine kidney cortex. J Am Chem Soc.

[4] Maret W (2011). Redox biochemistry of mammalian metallothioneins. J Biol Inorg Chem.

[5] Zangger K, Oz G, Haslinger E, Kunert O, Armitage I (2001). Nitric oxide selectively releases metals from the amino-terminal domain of metallothioneins: potential role at inflammatory sites. FASEB J.

[6] Li Y, Cam J, Bu G (2001). Low-density lipoprotein receptor family: endocytosis and signal transduction. Mol Neurobiol.

[7] Spuch C, Ortolano S, Navarro C (2012). LRP-1 and LRP-2 receptors function in the membrane neuron. Trafficking mechanisms and proteolytic processing in Alzheimer’s disease. Front Physiol.

[8] Chung RS, Penkowa M, Dittmann J, King CE, Bartlett C, Asmussen JW, Hidalgo J, Carrasco J, Leung YKJ, Walker AK, Fung SJ, Dunlop SA, Fitzgerald M, Beazley LD, Chuah MI, Vickers JC, West AK (2008). Redefining the role of metallothionein within the injured brain: extracellular metallothioneins play an important role in the astrocyte-neuron response to injury. J Biol Chem.

[9] Ambjørn M, Asmussen JW, Lindstam M, Gotfryd K, Jacobsen C, Kiselyov VV, Moestrup SK, Penkowa M, Bock E, Berezin V (2008). Metallothionein and a peptide modeled after metallothionein, EmtinB, induce neuronal differentiation and survival through binding to receptors of the low-density lipoprotein receptor family. J Neurochem.

[10] West AK, Leung JYK, Chung RS (2011). Neuroprotection and regeneration by extracellular metallothionein via lipoprotein-receptor-related proteins. JBIC J Biol Inorg Chem.

[11] Asmussen JW, Von Sperling ML, Penkowa M (2009). Intraneuronal signaling pathways of metallothionein. J Neurosci Res.

[12] Leung JYK, Bennett WR, Herbert RP, West AK, Lee PR, Wake H, Fields RD, Chuah MI, Chung RS (2012). Metallothionein promotes regenerative axonal sprouting of dorsal root ganglion neurons after physical axotomy. Cell Mol Life Sci.

[13] Landowski LM, Pavez M, Brown LS, Gasperini R, Taylor BV, West AK, Foa L (2016). Low-density lipoprotein receptor-related proteins in a novel mechanism of axon guidance and peripheral nerve regeneration. J Biol Chem.

[14] Jakovac H, Grubić Kezele T, Radošević-Stašić B (2018). Expression profiles of metallothionein I/II and megalin in cuprizone model of de-and remyelination. Neuroscience.

[15] Gumulec J, Raudenska M, Adam V, Kizek R, Masarik M (2014). Metallothionein - immunohistochemical cancer biomarker: a meta-analysis. PLoS One.

[16] Theocharis SE, Margeli AP, Klijanienko JT, Kouraklis GP (2004). Metallothionein expression in human neoplasia. Histopathol.

[17] Eckschlager T, Adam V, Hrabeta J, Figova K, Kizek R (2009). Metallothioneins and cancer. Curr Protein Pept Sci.

[18] Pedersen MO, Larsen A, Stoltenberg M, Penkowa M (2009). The role of metallothionein in oncogenesis and cancer prognosis. Prog Histochem Cytochem.

[19] Andersen R, Hammer K, Hager H, Christensen J, Ludvigsen M, Honoré B, Thomsen M, Madsen M (2015). Melanoma tumors frequently acquire LRP2/megalin expression, which modulates melanoma cell proliferation and survival rates. Pigment Cell Melanoma Res.

[20] Atrian S, Capdevila M (2013). Metallothionein-protein interactions. BioMol Concepts.

[21] Cherian MG, Jayasurya A, Bay BH (2003). Metallothioneins in human tumors and potential roles in carcinogenesis. Mutat Res.

[22] Ostrakhovitch EA, Olsson PE, von Hofsten J, Cherian MG (2007). P53 mediated regulation of metallothionein transcription in breast cancer cells. J Cell Biochem.

[23] Pedersen M, Hansen P, Nielsen S, Penkowa M (2010). Metallothionein-I + II and receptor megalin are altered in relation to oxidative stress in cerebral lymphomas. Leuk Lymphoma.

[24] Raleigh JA, Chou SC, Calkins-Adams DP, Ballenger CA, Novotny DB, Varia MA (2000). A clinical study of hypoxia and metallothionein protein expression in squamous cell carcinomas. Clin Cancer Res.

[25] Walentowicz-Sadlecka M, Koper A, Krystyna G, Koper K, Basta P, Mach P, Skret-Magierlo J, Dutsch-Wicherek M, Sikora J, Grabiec M, Kazmierczak W, Wicherek L (2013). The analysis of metallothionein immunoreactivity in stromal fibroblasts and macrophages in cases of uterine cervical carcinoma with respect to both the local and distant spread of the disease. Am J Reprod Immunol.

[26] McCluggage WG, Maxwell P, Bharucha H (1998). Immunohistochemical detection of metallothionein and MIB1 in uterine cervical squamous lesions. Int J Gynecol Pathol.

[27] Jemal A, Bray F, Center M, Ferlay J, Ward E, Forman D (2011). Global cancer statistics. CA Cancer J Clin.

[28] Jakovac H, Grebic D, Mrakovcic-Sutic I, Tota M, Broznic D, Marinic J, Tomac J, Milin C, Radosevic-Stasic B (2006). Metallothionein expression and tissue metal kinetics after partial hepatectomy in mice. Biol Trace Elem Res.

[29] Jawanjal P, Salhan S, Dhawan I, Rath G (2015). Comparative analysis of p53 and p21 proteins in normal cervix and HPV associated precancerous and cancerous lesions of cervix. J Anat Soc India.

[30] Manning B, Cantley L (2007). AKT/PKB signaling: navigating downstream. Cell.

[31] McCluggage WG, Maxwell P, Hamilton PW, Jasani B (1999). High metallothionein expression is associated with features predictive of aggressive behaviour in endometrial carcinoma. Histopathology.

[32] Dutsch-Wicherek M, Popiela TJ, Klimek M, Rudnicka-Sosin L, Wicherek L, Oudinet JP, Skladzien J, Tomaszewska R (2005). Metallothionein stroma reaction in tumor adjacent healthy tissue in head and neck squamous cell carcinoma and breast adenocarcinoma. Neuro Endocrinol Lett.

[33] Alvira-Botero X, Carro E (2010). Clearance of amyloid-β peptide across the choroid plexus in Alzheimer’s disease. Curr Aging Sci.

[34] Fitzgerald M, Nairn P, Bartlett CA, Chung RS, West AK, Beazley LD (2007). Metallothionein-IIA promotes neurite growth via the megalin receptor. Exp Brain Res.

[35] Auderset L, Landowski LM, Foa L, Young KM (2016). Low density lipoprotein receptor related proteins as regulators of neural stem and progenitor cell function. Stem Cells Int.

[36] Zelger B, Hittmair A, Schir M, ÖFner C, ÖFner D, Fritsch PO, Böcker W, Jasani B, Schmid KW (1993). Immunohistochemically demonstrated metallothionein expression in malignant melanoma. Histopathol.

[37] Weinlich G (2009). Metallothionein-overexpression as a prognostic marker in melanoma. G Ital Dermatol Venereol.

[38] Klaassen CD, Liu J, Diwan BA (2009). Metallothionein protection of cadmium toxicity. Toxicol Appl Pharmacol.

[39] Gajera CR, Emich H, Lioubinski O, Christ A, Beckervordersandforth-Bonk R, Yoshikawa K, Bachmann S, Christensen EI, Götz M, Kempermann G, Peterson AS, Willnow TE, Hammes A (2010). LRP2 in ependymal cells regulates BMP signaling in the adult neurogenic niche. J Cell Sci.

[40] Li Y, van Kerkhof P, Marzolo MP, Strous GJ, Bu G (2001). Identification of a major cyclic AMP-dependent protein kinase A phosphorylation site within the cytoplasmic tail of the low-density lipoprotein receptor-related protein: implication for receptor-mediated endocytosis. Mol Cell Biol.

[41] May P, Reddy Y, Herz J (2002). Proteolytic processing of low density lipoprotein receptor-related protein mediates regulated release of its intracellular domain. J Biol Chem.

[42] Wicher G, Larsson M, Svenningsen ÅF, Gyllencreutz E, Rask L, Aldskogius H (2006). Low density lipoprotein receptor-related protein-2/megalin is expressed in oligodendrocytes in the mouse spinal cord white matter. J Neurosci Res.

[43] Brown M, Ye J, Rawson R, Goldstein J (2000). Regulated intramembrane proteolysis: a control mechanism conserved from bacteria to humans. Cell.

[44] Lee MY, Shen MR (2012). Epithelial-mesenchymal transition in cervical carcinoma. Am J Transl Res.

[45] Stewart CJR, McCluggage WG (2013). Epithelial–mesenchymal transition in carcinomas of the female genital tract. Histopathol.

[46] Christensen EI, Birn H, Storm T, Weyer K, Nielsen R (2012). Endocytic receptors in the renal proximal tubule. Physiology (Bethesda).

[47] Moestrup S, Verroust PJ (2001). Megalin- and cubilin-mediated endocytosis of protein-bound vitamins, lipids, and hormones in polarized epithelia. Annu Rev Nutr.

[48] Binnewies M, Roberts EW, Kersten K, Chan V, Fearon DF, Merad M (2018). Understanding the tumor immune microenvironment (TIME) for effective therapy. Nat Med.

[49] Bruno A, Mortara L, Baci D, Noonan DM, Albini A (2019). Myeloid derived suppressor cells interactions with natural killer cells and pro-angiogenic activities: roles in tumor progression. Front Immunol.

[50] Mocchegiani E, Giacconi R, Cipriano C, Malavolta M (2009). NK and NKT cells in aging and longevity: role of zinc and metallothioneins. J Clin Immunol.

