# Supplementary material for: Expression profiles of metallothionein-I/II and megalin/LRP-2 in uterine cervical squamous lesions
Source: Virchows Arch. 2020 Oct 21;478(4):735–46. doi: 10.1007/s00428-020-02947-w (PMC7990851; doi:10.1007/s00428-020-02947-w)
Supplement: Supplementary file 1 — (DOCX 14 kb) [file 428_2020_2947_MOESM1_ESM.docx]

***Table 1.* Antibodies used in this study**

| \| \|  \| **Code** \| **Host** \| **Dilution** \| **Source** \| \| --- \| --- \| --- \| --- \| --- \| \| ***Primary antibodies*** \| \| anti-MT I+II IgG1  (clone E9) \| MO639 \| mouse \| 1:50 \| Dako Cytomation, USA \| \| anti-megalin IgG  (H-245) \| sc-25470 \| rabbit \| 1:200 \| Santa Cruz Biotechnology, USA \| \| anti-CD3 IgG \| ab5690 \| rabbit \| 1:100 \| Abcam, UK \| \| anti-AKT1  (phospho T308) IgG1  (clone 18F.3.H11) \| ab105731 \| mouse \| 1:50 \| Abcam, UK \| \| ***Secondary antibodies*** \|  \|  \|  \|  \| \| Alexa Fluor 555- labeled anti-mouse IgG \| A32727 \| goat \| 1:500 \| Thermo Fisher Scientific, USA \| \| Alexa Fluor 488-labeled anti-rabbit IgG \| A21206 \| donkey \| 1:300 \| Thermo Fisher Scientific, USA \| \| \| --- \| --- \| --- \| --- \| --- \| --- \| --- \| --- \| --- \| --- \| --- \| --- \| --- \| --- \| --- \| --- \| --- \| --- \| --- \| --- \| --- \| --- \| --- \| --- \| --- \| --- \| --- \| --- \| --- \| --- \| --- \| --- \| --- \| --- \| --- \| --- \| --- \| --- \| --- \| --- \| --- \| --- \| |
| --- | --- | --- | --- | --- | --- | --- | --- | --- | --- | --- | --- | --- | --- | --- | --- | --- | --- | --- | --- | --- | --- | --- | --- | --- | --- | --- | --- | --- | --- | --- | --- | --- | --- | --- | --- | --- | --- | --- | --- | --- | --- | --- |
