# Supplementary material for: Expression profiles of metallothionein-I/II and megalin/LRP-2 in uterine cervical squamous lesions
Source: Virchows Arch. 2020 Oct 21;478(4):735–46. doi: 10.1007/s00428-020-02947-w (PMC7990851; doi:10.1007/s00428-020-02947-w)
Supplement: Supplementary file 2 — (DOCX 15 kb) [file 428_2020_2947_MOESM2_ESM.docx]

***Table 2.* Expression scores of MT-I/II in LSIL and HSIL**

|  | Intact cervix | LSIL | HSIL | |
| --- | --- | --- | --- | --- |
|  | Normal | CIN1 | CIN2 | CIN3/CIS |
| Mean ± SE Grade (%) | | | | |
| Basal and parabasal epithelial cells | 1±0  Absent (100%) | 2.0±0.5  Low (88%) | 8.3±2.3  High (87.0%) | 7.3±2.7  High (93.0%) |
| Higher layers of dysplastic epithelium | 0±0  Absent (100%) | 0.9±0.6  Absent (84%) | 7.3±2.7  High (87.0%) | 1.9±1.3  Low (40%) |
| Cytoplasmic  expression | 0.6±0.5  Absent (100%) | 2.5±1.1 (88%)  Low | 6.5±3.6  High (66.7%) | 5.3±2.2  High (73.3%) |
| Nuclear expression | 0.2±0.4  Absent (100%) | 0.5±0.8  Absent (84%) | 6.0±1.4  High (80.0%) | 5.5±2.2  High (53.3%) |
| Mononuclear lymphatic cells | 0±0  Absent (100%) | 0.1±0.2  Absent (100%) | 5.2±1.2  High (66.7%) | 6.1±1.7  High (73.3%) |
|  |  |  |  |  |
| Number of cases | 5 | 25 | 15 | 15 |

Total scores of MT I+II immunoreactivities were obtained by multiplying the staining intensity (0-3) by percentage of positive cells (0-4) in areas of normal/dysplastic squamous epithelia and adjacent stroma. According to the product the resulting scorings were then classified into three grades: 0-1=absent, 2-5=low expression, and 6-12=high expression (shown in blue). In parenthesis is presented the number of affected cases in percentage.
