# Supplementary material for: Expression profiles of metallothionein-I/II and megalin/LRP-2 in uterine cervical squamous lesions
Source: Virchows Arch. 2020 Oct 21;478(4):735–46. doi: 10.1007/s00428-020-02947-w (PMC7990851; doi:10.1007/s00428-020-02947-w)
Supplement: Supplementary file 3 — (DOCX 15 kb) [file 428_2020_2947_MOESM3_ESM.docx]

***Table 3.* Expression scores of megalin in LSIL and HSIL**

|  | Intact cervix | LSIL | HSIL | |
| --- | --- | --- | --- | --- |
|  |  | CIN1 | CIN2 | CIN3/CIS |
|  | Mean ± SE Grade (%) | | | |
| Basal and parabasal epithelial cells | 0±0  Absent (100%) | 1.5±0.4  Absent (52%) | 7.1±2.3  High (80%) | 6.1±2.6  High (60.0%) |
| Higher layers of dysplastic epithelium | 0±0  Absent (100%) | 0.3±0.4  Absent (100%) | 6.0±1.4  High (46.7%) | 5.0±0.9  High (53.3%) |
| Cytoplasmic  expression | 0±0  Absent (100%) | 1.7±0.8  Absent (40%) | 4.9±1.2  High (53.3%) | 5.4±1.8  High (66.7%) |
| Nuclear expression | 0±0  Absent (100%) | 0.9±1.1  Low (40%) | 5.8±1.7  High (86.7%) | 5.6±1.6  High (73.3%) |
| Glandular epithelium | 0±0  Absent (100%) | 0.6±0.8  Absent (72%) | 6.5±1.3  High (93.3%) | 6.6±1.0  High (100%) |
| Mononuclear lymphoid cells | 0±0  Absent (100%) | 0.6±0.8  Absent (80%) | 6.0±2.3  High (80%) | 6.5±2.1  High (66.7%) |
| Number of cases | 5 | 25 | 15 | 15 |

Total scores of megalin immunoreactivities were obtained by multiplying the staining intensity (0-3) by percentage of positive cells (0-4) in areas of normal/dysplastic squamous epithelia and adjacent stroma. According to the product the resulting scorings were then classified into three grades: 0-1=absent, 2-5=low expression, and 6-12=high expression (shown in blue). In parenthesis is presented the number of affected cases in percentage.
