# Supplementary figures and images for: Expression profiles of metallothionein-I/II and megalin/LRP-2 in uterine cervical squamous lesions
Source: Virchows Arch. 2020 Oct 21;478(4):735–46. doi: 10.1007/s00428-020-02947-w (PMC7990851; doi:10.1007/s00428-020-02947-w)

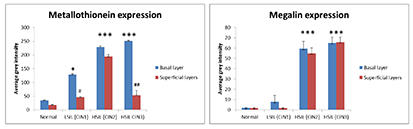

Supplement: Supplementary file 4 — Supplement. MT-I/II and megalin expression in cervical squamous epithelium in different SIL/CIN categories. The intensity and distribution of MT and megalin staining were estimated by ImageJ software analysis. The data are expressed as mean grey value ± SE. *p < 0.05, **p < 0.01 and ***p < 0.001 in comparison with basal layer in intact cervix; #p < 0.05, ##p < 0.01 comparison of basal and superficial layer in each group (PNG 158 kb) [file 428_2020_2947_Fig6_ESM.png]

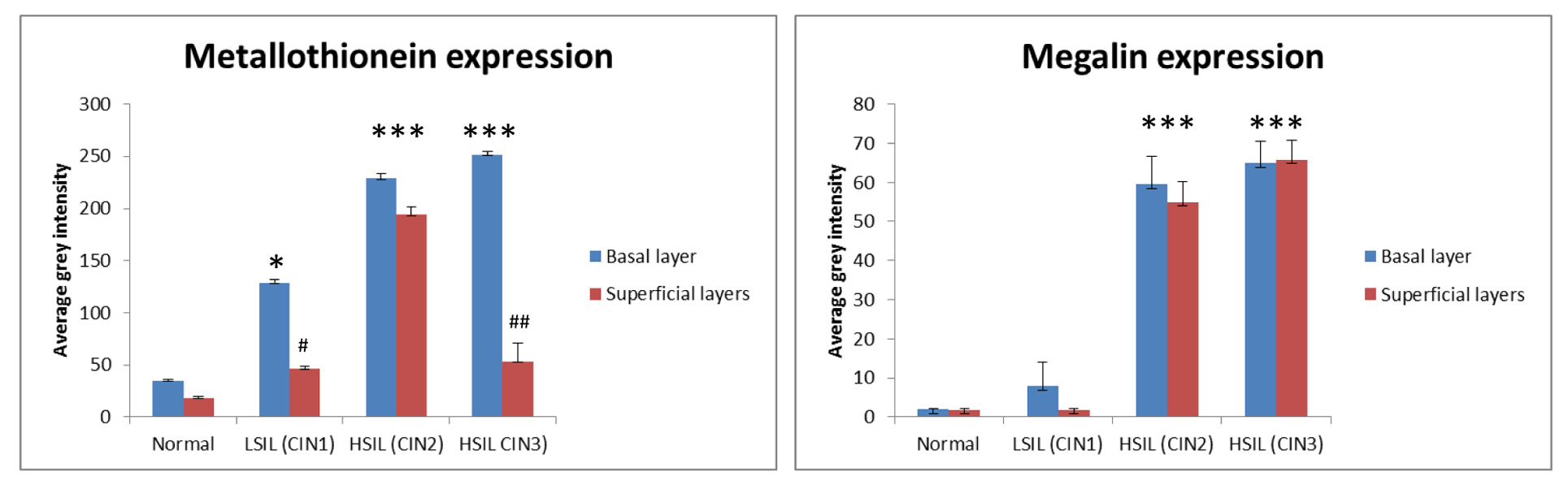

Supplement: Supplementary file 5 — High resolution image (TIF 183 kb) [file 428_2020_2947_MOESM4_ESM.tif]
